# The Role of Infarct Border Zone Remodelling in Ventricular Arrhythmias: Bridging Basic Research and Clinical Applications

**DOI:** 10.1111/jcmm.70526

**Published:** 2025-03-30

**Authors:** Jin Ma, Qiuxiong Chen, Dongqun Lin, Shiyu Ma

**Affiliations:** ^1^ Guangdong Provincial Hospital of Chinese Medicine The Second Affiliated Hospital of Guangzhou University of Chinese Medicine Guangzhou China; ^2^ State Key Laboratory of Dampness Syndrome of Chinese Medicine Guangzhou China

**Keywords:** cardiac remodelling, infarct border zone, myocardial infarction, sudden cardiac death, ventricular arrhythmias

## Abstract

Patients who experience post‐myocardial infarction (MI) and present with a left ventricular ejection fraction of less than 35% are classified as being at high risk for sudden cardiac death due to ventricular arrhythmias (VAs). The expansion of scar tissue and the extension of the infarct border zone (IBZ) following MI play critical roles in the progression of heart failure and the onset of VAs. Various aspects of structural remodelling, including cardiac fibrosis, along with electrophysiological changes such as alterations in gap junctions, ion channels, and autonomic nervous system function within the IBZ may contribute to abnormal impulse generation and conduction, thereby increasing susceptibility to arrhythmias. Currently, management strategies for VAs primarily encompass pharmacologic interventions (e.g., β‐blockers, amiodarone), device‐based approaches (e.g., ICD implantation), or catheter ablation techniques as outlined by ESC Guidelines. In this review, we systematically summarise both structural characteristics inherent in ischaemic myocardial substrates and clinical treatment strategies regarding VAs. We propose that early prevention strategies aimed at mitigating arrhythmogenic substrate formation represent an innovative approach to treating VAs following MI.

## 1 Introduction

Myocardial infarction (MI) is one of the most prevalent diseases globally, characterised by a high incidence of mortality and significant economic costs. Although advancements in MI treatment have enhanced survival rates following ischaemic cardiac injury, MI continues to account for over 15% of annual deaths. Cardiac remodelling after MI is a progressive process: once initiated, it leads to further deterioration of cardiac function or progresses to heart failure (HF). Patients with post‐MI and left ventricular (LV) ejection fraction (EF) < 35% are classified as a high‐risk group for sudden cardiac death (SCD), which can be precipitated by ventricular arrhythmias (VAs). Notably, 42.4% of patients who experienced SCD without any clinical history of coronary artery disease (CAD) were found at autopsy to have silent MI and myocardial scarring [1]. Consequently, SCD has recently been recognised as a significant public health concern [2]. Despite substantial reductions in the incidence of SCD attributable to advanced pharmacologic therapies and the introduction of implantable cardioverter‐defibrillator (ICD) devices, malignant arrhythmias remain a predominant cause of SCD among post‐MI patients.

Post‐MI structural and functional remodelling leads to the formation of vulnerable substrates that significantly increase the risk of VAs. LV remodelling following MI is a dynamic process that extends from days to years and is characterised by myocyte necrosis and programmed cell death, interstitial fibrosis, alterations in gap junctions, changes in ion channels, and modifications in the autonomic nervous system [3]. Each factor plays a critical role in the development and progression of HF and VAs [4]. This review will concentrate on the structural characteristics inherent in ischaemic myocardial substrates that contribute to the initiation of VAs along with clinical treatment strategies following healed MI. The strategy aimed at early prevention of arrhythmogenic substrate formation is expected to become an innovative therapeutic approach for preventing and treating VAs after MI.

## 2 Different Structural Regions in the Ventricle After MI

**FIGURE 1:**
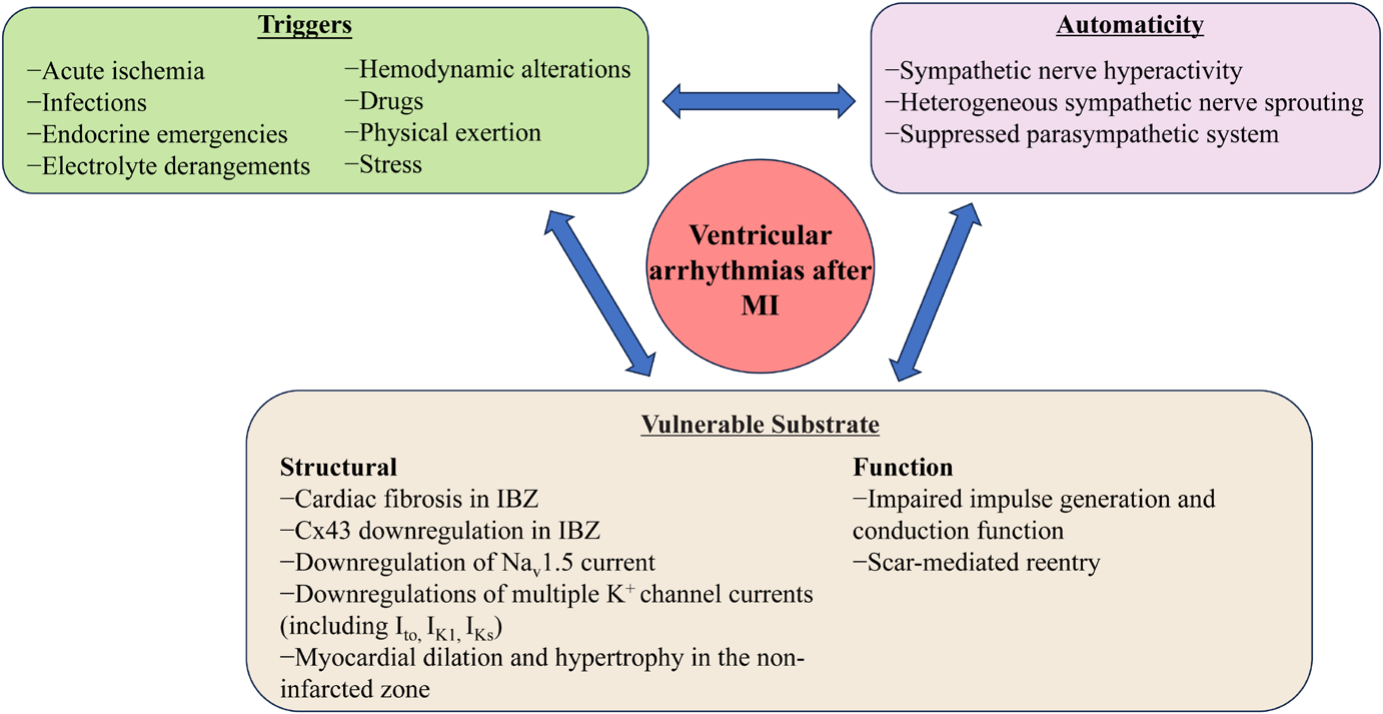
Overview of the fundamental electrophysiological mechanisms of VAs in post‐MI. Multiple mechanisms exist for VAs such as triggered activity, reentry, and automaticity. Scar‐based reentry is a predominant mechanism responsible for most malignant VAs that can ultimately lead to SCD. Some aspects of the structural (myocardial fibrosis and hypertrophy) and electrophysiological (altered gap junction, ion channel and autonomic nervous system) remodelling, especially in the IBZ may lead to impaired impulse generation and conduction. These changes can also significantly affect repolarisation dynamics and repolarisation reserve, rendering the heart more susceptible to arrhythmogenesis. Triggered ventricular activity such as acute ischaemia, infections, drugs and so on, generally develops after depolarisations due to oscillations in membrane potential after an action potential, which meet the threshold for triggering new ventricular action potentials. Cx, connexin; IBZ, infarct border zone; I_K1_, the inward rectifier K^+^ current; I_Ks_, the slow delayed rectifier K^+^ current; I_to_, the transient outward current; MI, myocardial infarction; SCD, sudden cardiac death; VAs, ventricular arrhythmias.

Following MI, the ongoing expansion and extension of infarcted scar into the border zone significantly contribute to the progression of HF and the occurrence of VAs [5, 6]. MI is a dynamic pathological process that commences with acute regional myocardial ischaemia, leading to myocardial injury or necrosis, and culminates with cardiac remodelling characterised by fibrosis. Cardiomyocyte death and inflammation are hallmark features during the early stage of MI [7, 8, 9]. In this phase, cardiomyocyte death occurs through both necrosis and programmed cell death, which triggers the release of cytokines that subsequently recruit and activate neutrophils, monocytes‐macrophages and fibroblasts [10]. Continued leukocyte recruitment and inflammation, along with the replacement of damaged cardiomyocytes by collagenous scar tissue, result in the formation of a scar zone. This collagen‐based scar tissue plays a crucial mechanical role by protecting against cardiac rupture while limiting dyskinetic bulging post‐healing [11]. However, it is important to note that scar tissue does not have the same structure or function as normal myocardial tissue [12]. Specifically, fibrous scars lack two essential functions inherent to normal myocardial tissue: they cannot contract rhythmically, nor do they efficiently conduct electrical signals [13]. Furthermore, surviving cardiomyocytes adapt to the altered conditions resulting from loss within part of the ventricle. Infarcted hearts undergo significant remodelling processes that include compensatory dilation and myocardial hypertrophy in non‐infarcted zones (Figure 1); these changes ultimately provide a substrate conducive to cardiac failure and arrhythmia development [14].

Remodelling following MI results in the formation of three distinct structural regions. The first region is the infarct zone, also known as the scar zone, which is typically subjected to ischemia. In this area, necrotic tissue is replaced by fibrous tissue, leading to scar formation that helps maintain wall integrity [15]. The second region comprises normal myocardium (non‐infarcted zone), which remains unaffected by the ischemic insult [16]. The third region is the infarct border zone (IBZ), characterised by a thin layer of surviving myocardium interspersed with fibrous tissue surrounding the scar [11]. In contrast to other regions, fibrosis within the IBZ exhibits an irregular and fragmented distribution, resulting in increased obstacles to conduction velocity [17]. This border zone is frequently targeted for catheter ablation procedures aimed at treating monomorphic VAs through high‐density voltage mapping. High‐density voltage mapping is a three‐dimensional reconstruction technique that utilises high‐density electrode arrays for assessing cardiac electrical activity. Through this advanced mapping technique, clinicians can more accurately identify and locate the origins of ventricular arrhythmias, thereby improving both the success rate and safety of ablation therapy.

## 3 Cardiac Remodelling in the IBZ as a Vulnerable Site for VAs

VAs are prevalent among patients with underlying cardiac structural abnormalities, including coronary heart disease, HF, myocardial hypertrophy, ion channel diseases, and conduction abnormalities. These conditions create a vulnerable substrate conducive to arrhythmia propagation. Various pathological factors have been identified that induce both adaptive and maladaptive changes in the structure, metabolism, and electrical function of the heart. Collectively referred to as “cardiac remodelling”, these alterations encompass specific aspects of structural remodelling such as fibrosis and myocardial hypertrophy, and electrophysiological remodelling including modifications in gap junctions, ion channels, and autonomic nervous system activity (Figure 1). Such abnormalities are particularly pronounced within the IBZ and can lead to impaired impulse generation and conduction alongside significant alterations in repolarization dynamics and repolarization reserve; consequently increasing the risk of arrhythmias (Figure 1).

### 3.1 Cardiac Fibrosis as a Determinant Substrate for VAs

Post‐MI ventricular remodelling is primarily characterised by scar formation accompanied by varying degrees of interstitial fibrosis within the IBZ that facilitate arrhythmogenesis through reentry mechanisms. Ding et al. found that both the size of the infarcted scar and the level of cardiac fibrosis in the IBZ were continuously increased after MI. At 28 days post‐MI, there was nearly a 30% increase in infarcted scar size and a 15% increase in fibrotic area within the IBZ [18]. Replacement myocardial fibrosis occurs following cardiac injury, particularly after MI, triggering a cascade of responses wherein necrotic cardiomyocytes are replaced by collagen‐based scars [19]. The quality of post‐MI remodelling is influenced not only by the size of the scar zone but also by qualitative characteristics inherent to the reparative response. Inadequate repair may result in low tensile strength scars, leading to chamber dilation and systolic HF. Conversely, persistent activation of myofibroblasts within the IBZ drives progressive fibrotic remodelling, which significantly contributes to deteriorating cardiac function along with conduction dysfunction [20]. An excess deposition of extracellular matrix (ECM) proteins in the myocardium leads to the electrical decoupling among myocytes, which affects both impulse generation and conduction. Ding reported that the conduction velocity of the MI border zone decreased to 53% compared to normal areas remote from the infarct site [21]. Therefore, cardiac fibrosis serves as a critical substrate and can be considered a predictor for VAs in patients with an infarcted heart (Figure 1) [22].

**FIGURE 2:**
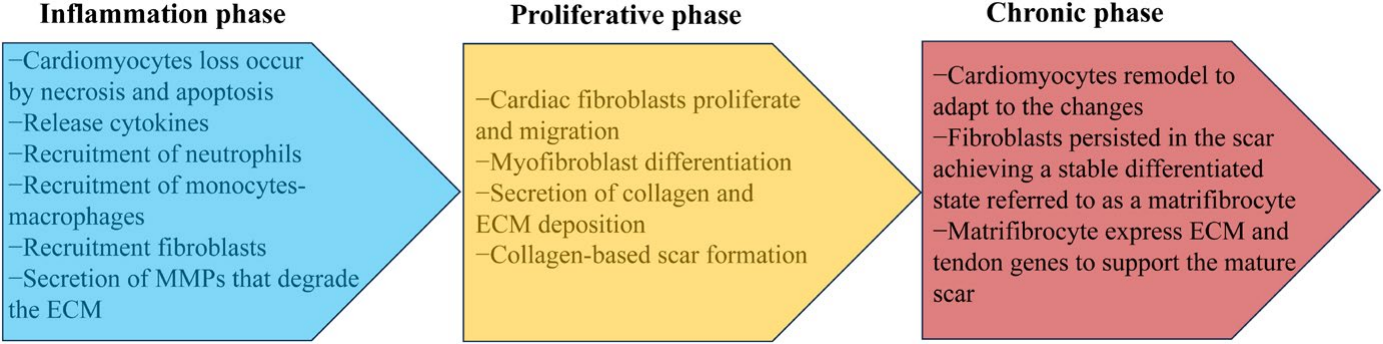
Phenotypic transitions of cardiac fibroblasts in the infarcted myocardium. During the inflammatory phase of infarct healing, necrotic cardiomyocytes undergo necrosis and apoptosis, releasing damage‐associated molecular patterns that activate a pro‐inflammatory response, such as IL‐1β, TNF‐α, and IL‐6. These cytokines stimulate the expression and secretion of MMPs that degrade the ECM, setting the stage for replacing damaged tissue with a collagen‐based scar. Resident cardiac fibroblasts do not express the myofibroblast marker α‐SMA. Matrix‐degrading phenotypes in cardiac fibroblasts contribute to the infiltration and activation of immune cells and fibroblasts at the injury site. The clearance of dead cardiomyocytes stimulates anti‐inflammatory signals, leading to the transition to the proliferative phase of infarct healing. During the proliferative phase, many fibroblasts become activated, proliferate, and differentiate into cardiac myofibroblasts that secrete large amounts of ECM proteins. In the maturation phase of infarct healing, fibroblasts display the disassembly of stress fibres and transition to matrifibrocytes, specialised cells that may play a role in scar maintenance. α‐SMA, α‐smooth muscle actin; ECM, extracellular matrix; IL, interleukin; MMPs, matrix metalloproteinases; TNF, tumour necrosis factor.

Activated fibroblasts and myofibroblasts serve as key mediator cells in cardiac fibrosis and pathological remodelling. These cells represent the main source of ECM deposition at the cellular level [9]. Cardiac fibroblasts (CFs) undergo three distinct phases of dramatic phenotypic transitions following MI (Figure 2) [9, 23]. (1) In the inflammatory phase, necrotic cardiomyocytes release signals that initiate an inflammatory response and promote a matrix‐degrading phenotype, which may facilitate the recruitment of leukocytes to the injury site. The removal of dead cardiomyocytes stimulates anti‐inflammatory signals, leading to a transition into the proliferative phase of infarct healing. (2) During the proliferative phase, resident fibroblasts from non‐infarcted regions proliferate and migrate, resulting in an increased population of fibroblasts within the infarct area. A significant proportion of these fibroblasts differentiate into myofibroblasts, characterised by high cellular contractility and substantial synthesis of ECM proteins. Various cytokines—including interleukin (IL)‐1, IL‐4, IL‐6, IL‐10, and tumour necrosis factor‐alpha—as well as growth factors such as platelet‐derived growth factor (PDGF) and transforming growth factor‐beta (TGF‐β), neurohumoral mediators like the renin‐angiotensin–aldosterone system (RAAS), mineralocorticoid receptors, β2‐adrenergic receptors, along with matricellular proteins have been implicated in activating fibroblasts and myofibroblasts [9]. CFs are also capable of sensing mechanical stress via integrins, ion channels, and mechanosensitive receptors. When pressure overload occurs, these mechanisms trigger intracellular fibrogenic cascades, which in turn lead to fibrosis. Myofibroblasts have a strong ability for secretion and proliferation, playing a key role in the deposition of ECM. (3) During the maturation phase, fibroblasts exhibit signs of disassembling stress fibres and transitioning into specialised cells known as matrifibrocytes, which may play a role in scar maintenance. Matrifibrocytes located in the mature scarred regions of human or murine hearts demonstrate a lack of EdU uptake capacity and diminished expression of α‐SMA [24]. These cells also resist further rounds of proliferation when exposed to Ang II/PE; conversely, fibroblasts in uninjured areas of the heart display significant proliferative activity [25]. Matrifibrocytes share certain gene signatures with chondrocytes and osteoblasts, both adapted to highly collagenous environments for constructing and maintaining cartilage and bone ECM. However, clustered gene analysis suggests that these cells are most closely related to quiescent fibroblasts [24]. Further mechanistic investigations are warranted to explore their potential as a strategy for positively influencing scar formation and long‐term remodelling following myocardial infarction.

Fibrosis is associated with an increased risk of arrhythmias and SCD. Strategies that involve the use of renin‐angiotensin‐aldosterone system (RAAS) inhibitors, such as angiotensin‐converting enzyme inhibitors (ACEIs), angiotensin II receptor blockers (ARBs), and mineralocorticoid receptor antagonists (MRAs), not only reduce fibrosis but also help to minimise individual susceptibility to arrhythmias [26, 27, 28]. There are several promising future directions for directly targeting the myocardial fibrogenic process in animal models. Renal denervation has been shown to reduce myocardial fibrosis and suppress VAs in a rat model of ischaemic cardiomyopathy [29]. Additionally, pirfenidone, a TGF‐β1 inhibitor, mitigated left ventricular fibrosis and decreased susceptibility to VAs in a rat model of MI [30]. Furthermore, certain drugs—such as vericiguat, muscone and relaxin—have demonstrated efficacy in reducing the risk of VAs in post‐MI animals. Research indicates that these agents possess not only anti‐fibrotic properties but also influence ion channels or gap junction proteins following MI [31, 32, 33]. Activated fibroblasts serve as key cellular effectors in matrix remodelling and reparative responses; thus, they represent potential therapeutic targets. Studies utilising animal models have suggested that modulating fibroblast functions may prevent adverse remodelling after MI [20, 34]. Although preclinical data on anti‐fibrotic compounds appear promising, their clinical application remains limited; therefore, further studies are warranted. Anti‐fibrotic treatment may emerge as an effective strategy for anti‐arrhythmic therapy following MI.

### 3.2 Gap Junction Remodelling in VAs

Gap junctions are composed of arrays of intercellular channels that facilitate the direct transfer of ions, second messengers, and small signalling molecules. These functions are crucial for maintaining homeostasis and ensuring synchronised excitation within cardiac tissue. The formation of these channels occurs through the oligomerization of protein subunits known as connexins (Cx) [35]. Similar to other tissues, the heart expresses various isoforms of Cx. Specifically, it has been identified that the heart expresses Cx30.2, Cx40, Cx43, and Cx45; these isoforms create cell‐to‐cell conduction pathways that mediate current flow essential for propagating cardiac impulses and regulating normal cardiac rhythm [36]. Remodelling of gap junctions is a prevalent pathological alteration observed in heart diseases associated with arrhythmias [37]. Alterations in gap junctional intercellular communication—critical for contact between adjacent cells and electrical coupling—can significantly affect overall cardiac function. There are two primary gating mechanisms involved: conventional membrane voltage‐dependent gating and transjunctional voltage‐dependent gating. Additionally, gap junction functionality is modulated by factors such as phosphorylation status, calcium (Ca^2+^) levels, pH variations, and the surrounding lipid environment. Exogenous agents—including peptides that enhance ionic conductance or lipophilic compounds that disrupt coupling—can also influence gap junction expression. In response to various pathological stressors, alterations in gap junction expression may occur, leading to abnormal cardiac conduction patterns and an increased risk of rhythm disturbances [38].

A growing number of research underscores the importance of gap junctions in relation to action potential conduction and the abnormalities that contribute to ventricular arrhythmogenesis. Following MI, gap junction remodelling alters their distribution and function, exacerbating conduction heterogeneity. The downregulation of Cx43 leads to a decrease in conduction velocity, creating a substrate for arrhythmia, which results in slower conduction and an increased risk of arrhythmias post‐MI (Figure 1) [39]. Studies have shown that modulating the coupling of gap junctions can influence the intercellular transfer of cell necrosis products, affect infarct progression, and potentially exert minor effects on the size of the healed infarct. Enhancing gap junction coupling during periods when natural uncoupling occurs is anticipated to facilitate the exchange of chemical mediators related to cell death and survival between healthy and dying cells at the IBZ during MI [40]. Rotigaptide has been demonstrated to enhance myocardial gap‐junction communication, increase ventricular conduction velocity, delay the onset of spatially discordant alternans, and reduce pacing‐induced VF risk during therapeutic hypothermia [41, 42, 43]. Furthermore, enhancing gap junction coupling with rotigaptide during acute MI resulted in more homogeneous scarring within the IBZ while also reducing late post‐MI VAs risk [41, 43]. Therefore, modulation of gap junctions following MI may represent a novel therapeutic strategy aimed at modifying healed infarct scar morphology and mitigating arrhythmic risk.

### 3.3 Ion Channels Remodelling in VAs

A trial conducted on a Chinese population has identified that ion‐channel genes KCNQ1, KCNH2, and SCN5A may play a significant role in the pathogenesis of VAs associated with MI [44]. Numerous cardiac ion channels are downregulated in ischaemic cardiomyopathy, and this downregulation contributes to the development of reentrant arrhythmias [45]. For example, a decrease in Na^+^ current density leads to reduced conduction velocity, thereby compromising impulse conduction within heart tissue adjacent to non‐infarcted myocardium located in the non‐ ischaemic risk zone (Figure 1) [46]. The downregulation of repolarizing K^+^ currents can prolong action potential duration (APD) and alter its dynamics. A diminished repolarization reserve is a critical electrophysiological consequence of K^+^ current downregulation, rendering the ventricular myocardium more susceptible to early afterdepolarizations and functional reentry phenomena [47]. Studies involving both human subjects and animal models have demonstrated that reductions in various K+ channel currents—including transient outward current (I_to_), inward rectifier K^+^ current (I_K1_), and slow delayed rectifier K^+^ current (I_Ks_)—can lead to an extension of APD (Figure 1) [48]. These reductions have been associated with decreased transcriptional activity, translational processes, and expression levels of relevant channels such as the slowly inactivating K^+^ channel (KvLQT1), inward rectifier K^+^ channel (Kir2.1), and rapidly inactivating K^+^ channel (Kv4.3).

Current anti‐arrhythmic medications inhibit ion channel activity and pose a significant proarrhythmic risk in patients with structural heart disease during clinical use. Several clinical trials, such as the Cardiac Arrhythmia Suppression Trials I and II, were abruptly terminated due to their inability to demonstrate a discernible level of protection for individuals suffering from severe arrhythmias [49]. Recent research indicates that ion channel‐blocking medications do not exhibit proarrhythmic potential when employed to prevent the downregulation of cardiomyopathy‐induced ion channels, thereby opening new therapeutic avenues. Transmembrane proteins are synthesised, folded, and assembled within the endoplasmic reticulum (ER) before being transported to the plasma membrane. These processes are critical for the proper functioning of cardiac ion channels and transporters. The unfolded protein response (UPR) serves as an essential mechanism for protein quality control in the ER; it monitors and regulates misfolded or unfolded proteins, ensuring correct protein folding and functionality within cells [50]. Activation of the Protein Kinase‐Like ER Kinase (PERK) branch of the UPR has been shown to increase arrhythmia risk through downregulation of specific cardiac ion channels following MI [51]. Pharmacological inhibition of PERK prevented reductions in Nav1.5, Kv4.3, and Kv1.5 channel proteins while resulting in shortened QTc intervals, fewer episodes of VAs, reduced mortality rates, and diminished overall arrhythmia risk in MI mouse models [52]. Investigations assessing the impact of PERK branch inhibition on arrhythmic risk post‐MI revealed that downregulation of ion channels during MI constitutes a fundamental mechanism underlying arrhythmogenesis. These findings suggest that targeting the PERK branch of the UPR represents a novel therapeutic strategy for antiarrhythmic intervention in ischaemic heart disease. The efficacy of this approach may support a broader hypothesis advocating that antiarrhythmic therapy should focus on preventing dysregulation of ion channels rather than solely blocking them.

The Nav1.5 current is encoded by the cardiac voltage‐gated sodium channel α subunit (SCN5A), which plays a critical role in cardiac electrical conduction and arrhythmogenic risk. Kang discovered that hypoxia increases the incidence of arrhythmia while decreasing Nav1.5 levels through its effect on miR‐448, whose expression is elevated in ischaemic cardiomyopathy. Following MI, inhibition of miR‐448 leads to an increase in Nav1.5 and reduces the likelihood of severe VAs [53]. Further, miR‐448 inhibition can restore KCNA4 expression during ischaemic conditions [54]. Zacopride, a selective IK1 agonist, has been shown to suppress the onset of VAs in MI rats by reversing the downregulation of Kir2.1 [55]. Additionally, LCZ696 (valsartan/sacubitril) therapy significantly decreased VA inducibility in post‐MI heart failure rats by upregulating potassium channel protein expressions such as ERG, KCNE1, and KCNE2 [56]. These studies further underscore that preventing ion channel downregulation represents a novel therapeutic strategy for antiarrhythmic treatment following MI.

### 3.4 Neural Remodelling in VAs

The sympathetic and parasympathetic branches of the autonomic nervous system collaboratively regulate heart function dynamically under healthy physiological conditions. However, in pathological cardiac states such as MI and HF, an imbalance occurs, characterised by increased sympathetic tone and decreased parasympathetic tone (Figure 1). This dysregulation arises from adverse remodelling within both the central nervous system and the cardiac neuraxis, which promotes disease progression and contributes to arrhythmogenesis [57]. Following MI, sympathetic fibres in scarred regions undergo degeneration and denervation, initiating a reinnervation process or nerve sprouting at the IBZ. Additionally, MI leads to a loss of efferent sympathetic innervation in non‐infarcted areas located distal or apical to the infarct site. Excessive activation of the sympathetic nervous system has been associated with an elevated risk of VAs in both human subjects and animal models. The hyperactivity of this system, along with uneven sprouting or hyperinnervation of sympathetic nerves, correlates with an increased incidence of VT and VF (Figure 1) [58]. Transmural MI disrupts the underlying myocardial substrate, resulting in a heterogeneous scar composed of thin myocyte fibres that conduct impulses slowly conditions—conducive to re‐entry mechanisms. This disruption also modifies the innervation patterns within histologically viable myocardium, further exacerbating arrhythmogenic potential. It is postulated that individuals suffering from MI and HF develop VAs due to a heterogeneous and hyperadrenergic electrophysiological response to sympathetic stimulation that increases dispersion during ventricular repolarisation. Arrhythmias have been effectively managed through pharmacologic interventions aimed at restoring this balance. By concentrating on the cardiac and extracardiac sympathetic nerves, various methods aimed at reducing sympathetic nerve activity have demonstrated efficacy in decreasing VAs. Specific clinical strategies, such as sympathetic ganglia blockade, cardiac sympathetic denervation, renal denervation and thoracic epidural anaesthesia, have reportedly been shown to reduce VAs by more than 80% in affected patients.

Neurotrophic factors play a major role in the pathophysiology of arrhythmias, innervation patterning and neuronal survival. Changes in the growth factors such as nerve growth factor (NGF) and semaphorin 3A (Sema3A) are involved in innervation patterning during the development of the neural remodelling following MI [59, 60]. Arrhythmias may arise from disrupted innervation or from the sprouting of sympathetic nerves. NGF promotes neural crest migration in developing neurons, enhancing neurite outgrowth and maturation. In addition to supporting cardiac neuronal survival, NGF regulates synapse formation and axonal growth during development. Although developed neurons continue to increase neurotransmitter synthesis, their dependence on NGF diminishes over time. The regeneration of cardiac sympathetic nerves and diverse patterns of innervation have been demonstrated by Zhou et al. who found elevated levels of NGF following MI in animal models [61]. Furthermore, according to Cao et al. NGF infusion during MI enhances myocardial nerve sprouting, resulting in a significant increase in SCD rates and a high incidence of ventricular arrhythmias. Sema3A functions as a potent neural chemorepellant that regulates axon and dendrite growth as well as neuronal migration. In the developing heart, Sema3A is highly expressed but progressively declines throughout development. It is believed that Sema3A acts as a negative regulator of cardiac sympathetic innervation by inhibiting neural growth, thereby contributing to the establishment of innervation patterns. In mice lacking Sema3A, sympathetic nerve density is observed to be higher in the subendocardium compared to lower levels found in the subepicardium. Evidence suggests that Sema3A suppresses sympathetic neuronal remodelling following MI [60, 62]. Research conducted by Chen et al. indicates that overexpression of Sema3A within the MI border zone may mitigate sympathetic hyperinnervation after an MI event; this reduction could subsequently decrease the inducibility of VAs [63].

## 4 Trigger Factors in VAs

The fundamental electrophysiological mechanisms underlying VAs encompass abnormal automaticity, triggered activity, and reentry (Figure 1). Triggered ventricular activity typically arises following depolarisations that reach the threshold necessary to induce new ventricular action potentials due to oscillations in membrane potential subsequent to an action potential. It is crucial to acknowledge the influence of trigger factors such as acute ischaemia, infections, pharmacological agents, haemodynamic fluctuations, endocrine emergencies, electrolyte imbalances, autonomic nervous system variations, physical exertion and psychological stress in precipitating SCD (Figure 1) [64, 65].

At the cellular level, calcium homeostasis has been proposed as a potential mechanism contributing to arrhythmia‐induced SCD. The sarcoplasmic reticulum (SR) serves a pivotal role in cardiac function by acting as the primary source of Ca^2+^ that activates cardiomyocyte contraction and contributes to triggers for cardiac arrhythmias and reentry phenomena [66]. Abnormal release of Ca^2+^ from the SR via ryanodine receptors (RyR2) has been implicated in arrhythmogenesis. Under conditions of high‐frequency stimulation, enhanced cytosolic Ca^2+^ buffering can lead to diminished Ca^2+^ transients and accumulation of Ca^2+^ during diastole. This process promotes spontaneous SR Ca^2+^ release and triggers activities such as early after‐depolarizations (EADs) and delayed after‐depolarizations (DADs) [67]. In hearts exhibiting failure characteristics, abnormalities in intracellular Ca^2+^ handling result in altered ventricular mechanics alongside increased electrical instability. Intracellular Ca^2+^ homeostasis is intricately linked with ventricular action potentials through mechanisms involving the Na^+^‐Ca^2+^ exchanger, calcium‐mediated inactivation of L‐type calcium channels, and activation of calcium‐sensitive transporters [68, 69]. Consequently, pharmacological modulation of cellular calcium handling has emerged as a focal point for both prevention and treatment strategies targeting VAs [70].

## 5 Cardiac Imaging in VAs

Transthoracic echocardiograms (TTEs) and coronary angiography remain the first‐choice diagnostic procedures for patients who present with VAs, according to current recommendations. Recent developments in imaging technology have made it possible to more precisely characterise the structural arrhythmogenic substrate in VA patients. Fibrosis is one of the most significant prognostic variables from a clinical standpoint [71, 72]. Cardiovascular magnetic resonance (CMR) imaging with late gadolinium enhancement (LGE) has emerged as the primary method for detecting cardiac fibrosis and has recently established itself as the in vivo gold standard for non‐invasive imaging of the structural substrate associated with VAs [73]. By measuring LGE signal intensity, CMR provides detailed visualisation of myocardial scars, comprehensive evaluations of cardiac architecture and function, along with exceptional soft‐tissue characterisation. Myocardial scars create regions that facilitate reentrant VAs, particularly within the IBZ, which disrupts electrical conduction [74]. While LGE is considered the most effective method for identifying focal fibrosis, diffuse fibrosis can be detected more accurately in its early stages through native T1 mapping and extracellular volume quantification using gadolinium contrast agents. Since its introduction, cardiac imaging has been utilised to guide interventional techniques aimed at treating VAs, thereby expanding its role from primarily diagnostic to adjunctive. Systems that integrate CMR imaging into mapping are currently under development. Furthermore, three‐dimensional (3D) systems have successfully facilitated safe and personalised radiofrequency ablation strategies based on substrate characteristics specifically for VAs [75]. 3D electroanatomic voltage mapping provides the capability to identify low‐voltage areas that correspond to regions of cardiomyocyte loss and collagenous replacement in injured hearts. The amplitudes of electrocardiograms are projected onto the anatomical shell at each point, facilitating the visualisation of scars, IBZ, and healthy tissues based on their respective maximal voltage amplitudes. A precise characterisation of these tissues is essential for identifying arrhythmogenic areas, enabling targeted ablation of potential future circuits, which fundamentally reduces the occurrence of arrhythmias.

## 6 Treatments for VAs in Clinic

**FIGURE 3:**
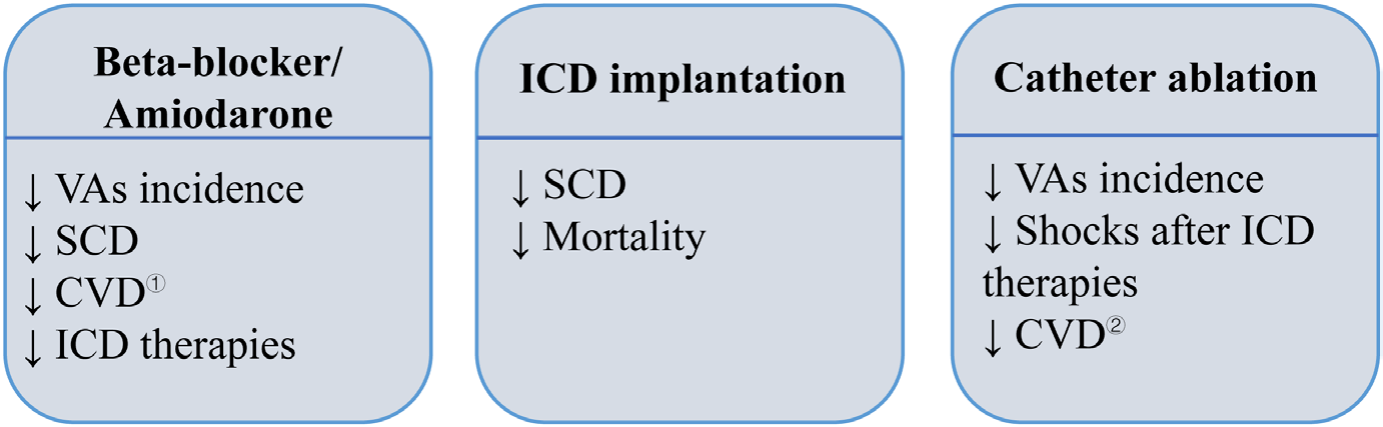
Prevention and treatment of VAs or SCD in patients with MI. ^①^Βeta‐blocker can reduce CVD events and improve long‐term prognosis. Amiodarone has no impact on CVD events. ^②^Early first‐line catheter ablation performed concurrently with ICD implantation probably reduces CVD events. CVD, cardiovascular disease; ICD, implantable cardioverter‐defibrillator; MI, myocardial infarction; SCD, sudden cardiac death; VAs, ventricular arrhythmias.

Patients with MI who undergo cardiac remodelling are at an increased risk of developing and exacerbating ventricular dysfunction, arrhythmias, and poor prognostic outcomes. It is essential to prevent acute deterioration and the progressive decline of underlying conditions while managing triggering activities; this constitutes a fundamental aspect of effective management strategies for VAs and the prevention of SCD. The European Society of Cardiology (ESC) Guidelines delineate three primary anti‐arrhythmic management approaches for VAs: pharmacological therapy, device‐based interventions, or interventional procedures (Figure 3) [76]. Amiodarone and β‐blockers serve as the cornerstone pharmacologic treatments for VAs. However, it is important to note that continuous use of β‐blockers may be associated with adverse side effects. An ICD represents a viable alternative in device‐based therapy. On the interventional front, catheter ablation currently stands as a leading approach [77].

Recent advancements in understanding the mechanistic underpinnings of arrhythmogenic substrates alongside technological innovations have facilitated enhanced interventional techniques that are undergoing clinical evaluation; however, progress in pharmacological therapies has lagged behind. Furthermore, advanced HF therapies such as left ventricular assist devices and heart transplantation may offer therapeutic options for select patients suffering from end‐stage HF with or without refractory VAs. One notable treatment modality is cardiac contractility modulation, which mitigates myocardial structural and electrical remodelling during HF by improving calcium handling and reversing fetal myocyte gene expression patterns associated with HF at the cellular level. This technology appears to be both effective and safe for treating HF along with its related complications [78, 79]. In addition, some promising therapeutic approaches such as the intramyocardial delivery of mesenchymal stem cells, extracellular vesicles, or platelet gel into the injured myocardium have been associated with a reduction in adverse left ventricular remodelling and preservation of cardiac function [78, 79, 80, 81]. Some studies have indicated that the injection of mesenchymal stem cells may reduce the risk of VAs following MI [82].

### 6.1 Pharmacologic Therapy

#### 6.1.1 β‐Blockers

β‐Blockers are still a mainstay for treating the majority of symptomatic VAs because they are safe, well‐tolerated, and beneficial for treating LV systolic dysfunction and symptomatic coronary heart disease [83]. β‐blockers were proven to be effective as an antiarrhythmic in avoiding VAs in prospective randomised studies. The β‐blockers propranolol and carvedilol have been demonstrated in numerous larger randomised controlled trials to be highly successful in lowering morbidity, mortality, and the incidence of SCD in addition to having an antiarrhythmic impact in post‐MI or HF (Table 1) [84, 85, 86, 87]. The mechanism of action of β‐blockers on VAs may involve impeding the release of excessive calcium through the ryanodine receptor channel and preventing a sympathetic trigger of VAs [88]. But an alarming discovery indicates that patients with more than two risk factors for shock were at higher risk of death when they were treated with β‐blockers [89]. Other adverse effects have been reported, such as hypotension, bradycardia, cold extremities, headaches, bronchospasm, increased insulin resistance, and insomnia [90].

**TABLE 1:** Randomised clinical trials which show that β‐blockers have an antiarrhythmic effect in preventing VAs.

Authors (refs)	Year	Drug name	Trial	Patients	Number	Control	Follow‐up (months)	Primary results
McMurray et al. [84]	2005	Carvedilol	CAPRICORN	Reduced left ventricular systolic function after MI	1959	Placebo	15.6	The rate of ventricular tachycardia/flutter/fibrillation were 38–984 (3.9%) in placebo group and 9–975 (0.9%) in carvedilol group
Packer et al. [85]	2002	Carvedilol	COPERNICUS	HF with an ejection fraction < 25% (but not volume‐overloaded)	2289	Placebo	10.4	The rate of ventricular tachycardia/fibrillation were 49–1133 (4.3%) in placebo group and 24–1156 (2%) in carvedilol group
Brodine et al. [86]	2005	β‐blockers	MADIT‐II	Previous MI and ejection fractions ≤ 30%	1232	Received ICDs	20	Patients receiving the top quartile of β‐blocker doses having significantly less use of ICD therapy for VT or VF than those not receiving β‐blockers
Goldstein [87]	1983	Propranolo	BHAT	Post‐MI	3837	Placebo	25	Propranolol therapy blunted the increase of ventricular arrhythmia at 6 weeks

Abbreviations: HF, heart failure; ICD, implantable cardioverter‐defibrillator; MI, myocardial infarction; VAs, ventricular arrhythmias; VF, ventricular fibrillation.

#### 6.1.2 Amiodarone

Amiodarone is a potent coronary and peripheral vasodilator that can be used safely in patients with LV dysfunction following MI, as well as those with congestive HF or hypertrophic cardiomyopathy. As a class III agent, it prolongs refractoriness in cardiac regions and prevents/terminates re‐entry, and it is rarely associated with QT interval prolongation and ventricular pro‐arrhythmia [91, 92]. Patients with HF who get amiodarone, especially if beta‐blockers are also taken, had a lower rate of arrhythmic mortality from VAs (see Table 2) [93, 94, 95, 96, 97]. However, it is unclear whether amiodarone improves the patients' overall and cardiovascular survival. Amiodarone is also a crucial adjuvant medication for lowering shocks in individuals who have an implanted cardioverter‐defibrillator [91]. Although amiodarone has a poor toxicity profile for long‐term use, it is nonetheless utilised to treat most instances of significant VAs, especially in the acute setting when hemodynamic disturbances are present [92]. A prior study demonstrated that when LVEF < 35%, amiodarone did not provide any more survival benefits than a placebo [98]. Its long‐term usage is limited by its antiarrhythmic activity, the significant risk of non‐cardiac toxicity, and frequent medication interactions. Therefore, amiodarone is generally considered a secondary therapeutic option [91].

**TABLE 2:** Amiodarone reduces the rate of VAs in patients with post‐MI or HF.

Authors (refs)	Year	Trial	Patients	Number	Control	Follow‐up	Primary results
Ceremuzynski et al. [93]	1992	−	High risk postinfarction patients	613	Placebo	12 months	A significant reduction in cardiac mortality and ventricular arrhythmias with amiodarone treatment
Navarro et al. [94]	1993	−	MI with a LVEF of 20%–45% and ≥ 3 ventricular premature complexes per hour	368	Metoprolol or no antiarrhythmic treatment	2.8 years	Long‐term treatment with amiodarone was effective in suppressing arrhythmias
Singh et al. [95]	1995	−	HF with an EF < 40%	674	Placebo	45 months	Amiodarone was significantly more effective in suppressing ventricular arrhythmias
Cairns et al. [96]	1997	CAMIAT	MI	1202	Placebo	1.79 years	Amiodarone reduces the incidence of VF or arrhythmic death among survivors of acute MI with frequent or repetitive VPDs
Watanabe et al. [97]	2018	SATISFACTION	Patients who had ICDs or who underwent cardiac resynchronization therapy with a defibrillator	612	−	11.3 ± 5.4 months	VAs/VF was controlled by amiodarone in all cases in the AF group but only in patients with an LVEF ≥ 40% in the SR group

Abbreviations: HF, heart failure; ICD, implantable cardioverter‐defibrillator; LVEF, left ventricular ejection fraction; MI, myocardial infarction; VAs, ventricular arrhythmias; VF, ventricular fibrillation; VPDs, ventricular premature depolarisations.

#### 6.1.3 Other Drug Therapy

After MI or in the presence of non‐ischaemic cardiomyopathy, the ventricle undergoes adverse remodelling. These structural modifications, along with related structural changes, may increase the risk of VAs. Due to an extension of the cardiac arrhythmia suppression trial (CAST) results, showing an excessive mortality or non‐fatal cardiac arrest rate (7.7%) among post–MI patients treated with encainide or flecainide compared with that in placebo‐treated patients (3.0%) which were reported three decades ago, many clinicians still commonly avoid treatment with Class IC antiarrhythmic drugs in patients with any known CAD. But in recent years, some studies indicate that in a limited population of MI patients with preserved left ventricle function, treatment with a 1C agent appears not to increase mortality [99, 100, 101]. Guidelines should include sotalol because it has been shown to be more effective than lidocaine in the acute termination of VAs [102]. ICD shocks have been reported to be reduced by sotalol, according to the optical pharmacological therapy in cardioverter‐defibrillator patients (OPTIC) trials [103].

No single drug has yet been found which can specifically target the susceptibility substrate of VAs‐myocardial remodelling in clinic. However, studies have found that some cardiovascular drugs also have the effect of improving myocardial remodelling and reducing the occurrence of arrhythmias. A number of medications, including ACEI, ARBs, and MRAs, decrease the rates of VAs and SCD and enhance reverse remodelling (Table 3) [104, 105, 106]. In high‐risk patients, these medications may lessen the incidence of potentially fatal VAs.

**TABLE 3:** Other drug therapy reduces the incidence of VAs in patients.

Authors (refs)	Year	Drug name	Patients	Number	Control	Follow‐up	Primary results
Connolly et al. [104]	2006	Sotalol	ICD patients	412	−	1 year	There was a trend for sotalol to reduce shocks compared with beta‐blocker alone
Askari et al. [105]	2009	ACEI/ARB	Acute MI patients	16,588	−	30 days	Baseline use of an ACEI/ARB was associated with a decreased incidence of early VF/VT
Wei et al. [106]	2010	Aldosterone antagonists (AAs)	Patients with HF or CAD	8635	Placebo/blank	2–24 months	The additional administration of AAs may be effective for reducing episodes of ventricular premature complexes and VA

Abbreviations: ACEI, angiotensin‐converting enzyme inhibitors; ARBs, angiotensin II receptor blockers; HF, heart failure; ICD, implantable cardioverter‐defibrillator; LVEF, left ventricular ejection fraction; MI, myocardial infarction; VAs, ventricular arrhythmias; VF, ventricular fibrillation.

### 6.2 Device Therapy

ICD implantation is recommended for the primary prevention of SCD in people with LVEF ≤ 35% and NYHA classes II–III (and life expectancy > 1 year), including those who have had a MI 40 days ago or a revascularization more than 3 months ago [107, 108]. Based on research showing a survival benefit with ICD implantation in systolic HF patients who are at risk of SCD, such as the Multicenter Automatic Defibrillator Implantation Trial I (MADIT I), MADIT II, MUSTT, and Sudden Cardiac Death in Heart Failure Trial (SCD‐HeFT) trials, these indications have been made [109]. ICD therapy is the only strategy that proved to reduce the mortality rate in preventing SCD in high‐risk individuals. However, it belongs to palliative treatment and cannot prevent or mitigate the risk of VAs occurrences or delay the progression of myocardial lesions. On the other hand, over the long run, defibrillators may cause complications, including inappropriate shocks, which have a 10% chance of inadvertent shock [110, 111]. Most significantly, ICDs are an inaccessible treatment in many nations due to their high upfront costs [112].

### 6.3 Interventional Therapy

Catheter ablation is the interventional technique used for VAs [113, 114]. Over the past 3 decades, important advancements have occurred in the understanding of the mechanisms of recurrent VAs, their prognostic implications in different clinical contexts, and their treatment options [115]. With the improvements in the safety and effectiveness of catheter ablation through technological and mechanistic advancements, ablation of VAs has steadily evolved from a last‐resort, palliative strategy to more upstream therapy. Now catheter ablation is a useful option for patients with recurrent, drug refractory monomorphic VAs and device therapy [116]. Catheter ablation is being increasingly performed as adjunctive treatment to prevent recurrent ICD therapies in patients with nonischemic cardiomyopathy and VAs. Tung et al. found among patients with cardiomyopathy and monomorphic VAs of varied causes, early catheter ablation performed at the time of ICD implantation significantly reduced the composite primary outcome of VAs recurrence and ICD shocks, cardiovascular hospitalisation, or death [117]. Catheter ablation for treating organic heart disease with VAs, which was previously considered a forbidden zone, has progressed to the stage at which ablation can improve prognosis after reducing the number of ICD shocks now.

## 7 Conclusion

Individuals with myocardial scars are at an elevated risk of SCD and life‐threatening arrhythmias. This review focused on the role of the structural characteristics of ischemic myocardium substrates in the development of VAs and treatment strategy in clinical practice following healed MI. Remodelling after MI results in three distinct structurally regions in the LV: the scar zone, the non‐infarcted zone and the IBZ. The expansion of the scar and the extension of the IBZ are key factors in the progression of HF and the occurrence of VAs after MI. Structural and electrophysiological changes including myocardial fibrosis, altered gap junction, ion channel and autonomic nervous system remodelling occur in the IBZ. While these changes may represent a cardiovascular adaptation to pathophysiological conditions, they can also lead to significant alterations in repolarization dynamics and repolarization reserve, thereby increasing susceptibility to arrhythmia. Researchers are dedicated to exploring new intervention methods for various susceptible substrates to reduce the risk of VAs.

Recent advancements in imaging technologies, such as CMR imaging with LGE and 3D electroanatomic voltage mapping, have enabled detailed characterisation of structural arrhythmogenic substrates within the heart. Currently, management strategies for VAs primarily encompass pharmacologic interventions (e.g., β‐blockers, amiodarone), device‐based approaches (e.g., ICD implantation), or catheter ablation techniques as outlined by ESC Guidelines. As technological progress enhances our understanding of MI pathophysiology's complex layers and its role as a vulnerable substrate for VAs, we gain opportunities to precisely target and modulate mechanisms that contribute to forming these arrhythmogenic substrates. Early intervention aimed at preventing substrate formation may emerge as a novel and effective therapeutic strategy for managing VAs subsequent to MI.

## Author Contributions

**Jin Ma:** conceptualization (equal), data curation (equal), investigation (equal), resources (equal), visualization (equal), writing – original draft (equal), writing – review and editing (equal). **Qiuxiong Chen:** funding acquisition (equal), supervision (equal). **Dongqun Lin:** supervision (equal). **Shiyu Ma:** funding acquisition (equal), supervision (equal), writing – original draft (equal), writing – review and editing (equal).

## Conflicts of Interest

The authors declare no conflicts of interest.

## Data Availability

The authors have nothing to report.
